# Postsystolic thickening is a potential new clinical sign of injured myocardium in marfan syndrome

**DOI:** 10.1038/s41598-021-95263-5

**Published:** 2021-08-04

**Authors:** Aleksandra Mas-Stachurska, Gustavo Egea, Rianne de Bruin-Bon, Paula Rudenick, Laura Sanchis, Berto J. Bouma, Barbara J. Mulder, Bart Bijnens, Marta Sitges

**Affiliations:** 1 Cardiovascular Institute, Hospital Clinic, University of Barcelona and Institut D’Investigacions Biomèdiques August Pi I Sunyer (IDIBAPS); CERCA Programme/Generalitat de Catalunya, Barcelona, Spain; 2 Institut Hospital del Mar d’Investigacions Mèdiques (IMIM), Barcelona, Spain; 3 Department of Biomedical Sciences, University of Barcelona School of Medicine and Health Sciences and Institut d’Investigacions Biomèdiques August Pi I Sunyer (IDIBAPS), Barcelona, Spain; 4 Cardiology Department, Amsterdam University Medical Centers, Location Academic Medic Centrum, Amsterdam, The Netherlands; 5 ICREA, Barcelona, Spain; 6 Cardiovascular Institute, Hospital Clinic, University of Barcelona School of Medicine and Health Sciences and Institut D’Investigacions Biomèdiques August Pi I Sunyer (IDIBAPS), CIBERCV, Instituto de Salud Carlos III (CB16/11/00354); CERCA Programme/Generalitat de Catalunya, Barcelona, Spain

**Keywords:** Ultrasound, Cardiomyopathies, Aortic diseases, Cardiology

## Abstract

The mechanisms leading to cardiac remodeling in Marfan syndrome (MFS) are a matter of debate since it could be either due to structural dysfunction of the myocardial extracellular matrix or to increased afterload caused by the dilated aorta. We aim to characterize the presence of abnormal myocardial function in MFS and to investigate its potential association with increased afterload. Aorta, left ventricle (LV) and the postsystolic thickening (PST) were analyzed in echocardiography in *Fbn1*^*C1039G/*+^ mice and in patients with MFS in comparison with wild type (WT) mice and healthy humans. PST was more frequent in MFS than in WT mice (p < 0.05). MFS mice with PST showed larger aorta than those without PST. Patients with MFS showed larger aorta, poorer LV function and a higher prevalence of PST (56%) than did the healthy controls (23%); p = 0.003. Blood pressure was similar. The higher prevalence of PST in an experimental murine model and in MFS patients, regardless of systemic arterial pressure, suggests an increased afterload on the LV myocardium. This finding supports the use of PST as an indicator of myocardial damage and encourage searching for novel early preventive therapy.

## Introduction

Marfan syndrome (MFS) is an autosomal dominant inherited disorder that affects skeletal, ocular and cardiovascular tissues. The main cardiovascular manifestations in MFS are ascending aorta dilation, especially localized at the level of the aortic root sinuses, aortic regurgitation and mitral valve prolapse with or without regurgitation^1^. Previous clinical^2–5^ and animal experimental^6^ studies in MFS have also reported intrinsic cardiac remodeling and mildly impaired ventricular function in the absence of significant valvular insufficiencies, raising the potential hypothesis of the existence of structural dysfunction of the extracellular matrix of the myocardium. Nonetheless, MFS patients with abnormal aortic wall could also be more susceptible to changes in pressure, showing more aortic wall stress and, consequently, increased myocardial afterload. The effect of pressure overload on the myocardial structure and function in MFS remains an open question.

Postsystolic thickening (PST) is an abnormal longitudinal myocardial thickening/shortening occurring after the closure of the aortic valve. It has been well described as a marker of segmental heterogeneity of loading or contractility, mainly in the setting of pressure overload and myocardial ischaemia^7–10^, and involves adverse functional myocardial remodeling. PST can be observed in the setting of altered loading conditions as hypertension in M-mode^7^, Tissue Doppler and 2D Speckle Tracking modalities of echocardiography. There is usually good correlation between those modalities in optimal conditions of experimental studies^11^. M-mode has the advantage of a higher temporal resolution which provides additional sensitivity to detect PST when image quality is limited or at situations of very initial stages of myocardial dysfunction.

We hypothesize that MFS patients may be more sensitive to blood pressure, despite their basal arterial pressures being in the normal range, and consequently they develop more segmental wall stress heterogeneity due to underlying abnormal arterial and/or myocardial wall tissue. Our purpose is to determine whether or not there is abnormal myocardial deformation in MFS and whether or not this deformation is related to typical altered regional deformation patterns that occur in response to increased afterload. As a first approach to this aim, we have chosen an experimental murine model of MFS mice (*Fbn1*^*C1039G/*+^ ), a well-established animal model of MFS^12^. In addition, an analysis has been conducted on a group of young humans affected from Marfan syndrome and healthy controls. We have examined the presence of PST in both experimental and clinical groups and its potential relationship with blood pressure and the development of aortic aneurysm.

## Methods

### Animal model

*Fbn1*^*C1039G/*+^ mice and wild type (WT) mice were obtained from Jackson Laboratory (Bar Harbor, ME 04,609, USA) and used as a validated MFS animal model. Both Wild Type (WT) and *Fbn1*^*C1039G/*+^ mice (hereafter, Marfan [MFS] mice) were bred on a C57BL/6 background. Comparisons were made between contemporary littermates. All mice were housed in a controlled environment (12/12-h light/dark cycle) and provided with ad libitum access to food and water. Animal care and experimentation conformed to the European Union (Directive 2010/63/EU) ARRIVE and Spanish guidelines (RD 53/2013) for the use of experimental animals. For the animal experiments, ethical approval was obtained from the local animal ethics committee (Ethics Committee on Animal Experimentation [CEEA], University of Barcelona).

The animal population consisted of two groups: group 1, consisting of 108 9 months (mo) age mice (53 MFS and 55 WT mice) in order to analyze the prevalence of PST as a marker of increased afterload in a large number of animals, and group 2, consisting of 39 4 mo-age mice (18 MFS and 21 WT mice) in a controlled study, with systematic determinations of arterial pressure in order to explore the association of PST with blood pressure levels.

Systemic blood pressure (BP) was noninvasively measured in the group of 4 mo-age mice by a tail cuff system (Panlab NIBP system, consisting of control unit LE5007 and the automatic heater and scanner for 6 mice, LE56506). Briefly, mice were placed in a warming/restraining box (34 °C), with the tail carefully inserted into an inflatable cuff. Systolic blood pressure (SBP) and diastolic blood pressure (DBP) were automatically measured. Before the final BP measurements, all mice were placed in the setup as many times as needed until they became adapted, thereby minimizing stress associated with the procedure.

### Clinical study patients

The group of Marfan patients consisted of 45 patients with established diagnoses of MFS by specialized cardiologists and according to the revised Ghent criteria. The healthy controls included 35 subjects examined by cardiac ultrasound as part of a familial screening for MFS disease and bicuspid aortic valve. Human subjects with more than mild valvular insufficiency or other significant forms of cardiomyopathy were excluded from the study. Clinical data was obtained from clinical records. The protocol was approved by the ethics committee of our institution (Medical Ethics Review Committee of the Academic Medical Center, reference number: W17_103 # 17.122app) in accordance with relevant guidelines and regulations. All participants gave informed consent. The data which supports the findings of this study is available upon reasonable request.

### Echocardiography and assessment of PST

A two-dimensional transthoracic echocardiogram was performed on all animals, mildly anesthetized, with 1.5% inhaled isoflurane^13^, and on conscious humans. Images were obtained from mice using a 10 to 13-MHz phased-array linear transducer (IL12i GE Healthcare, Milwaukee, US) with a Vivid Q system (GE Healthcare, Milwaukee, US) and from humans using a Vivid E9 (GE Healthcare, Oslo, Norway) with a transthoracic probe. All images were recorded and analyzed offline using commercially available software (EchoPac v. 108.1.6, GE Healthcare, Madrid, Spain). The aortic root and ascending thoracic aorta were measured in the parasternal long axis view. End-diastolic diameters were measured from inner to inner edge in mice and from leading edge to leading edge in humans at the aortic sinus level (for the aortic root) and at 1 mm and 1 cm above the sinotubular junction (for the ascending aorta) in mice and humans, respectively.

In the case of mice, an M-mode echocardiographic trace was acquired at the papillary muscle level in a parasternal short axis view, where LV dimensions at both end-diastole (LVDD) and end-systole (LVSD) were measured. The same M-mode trace was performed on humans in the basal parasternal long axis view. The interventricular septum and posterior wall thickness at end-diastole were measured at the same level as the LV chamber dimensions. LV ejection fraction (LVEF) was subsequently calculated as follows: LVEF = ((LVEDV−LVESV)/LVEDV)*100 where LVEDV = 7*LVEDD^3^/2.4 + LVEDD and LVESV = 7*LVESD^3^/2.4 + LVESD were computed by the Teichholz formula and used as surrogates for LV systolic function. LV mass was calculated from the Devereux formula for LV mass (g) = 0.8{1.04[([LVEDD + IVSd + PWd]3−LVEDD3)]} + 0.6.

The blood pressure value in human subjects was registered the day of the echocardiography examination. Body surface area (BSA) was calculated using the DuBois method: BSA = (W^0.425^ × H^0.725^) × 0.007184 where the weight (W) is in kilograms and the height (H) is in centimeters.

**Figure 1:**
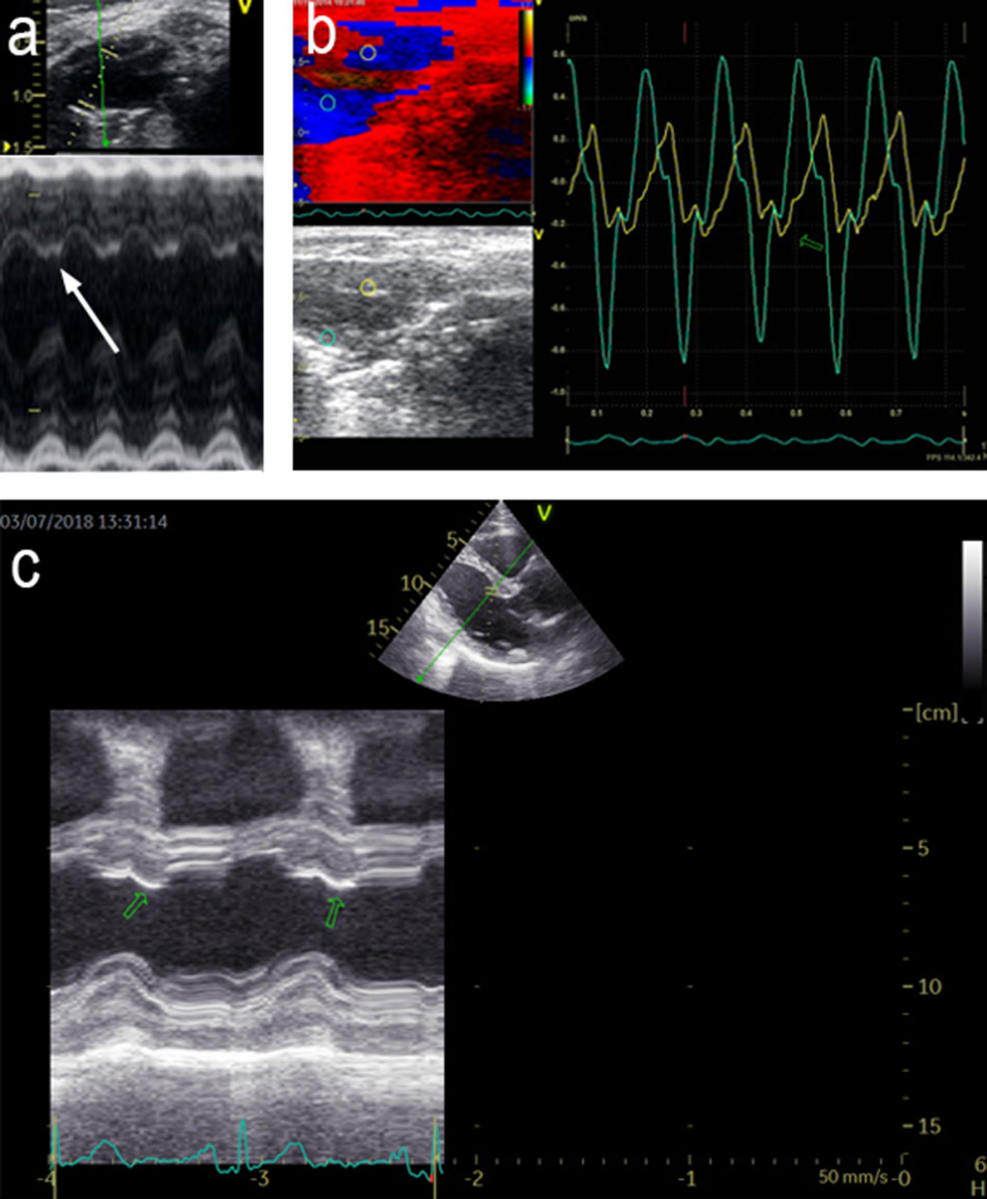
Echocardiographic assessment of PST. (**a**,**b**) Examples of PST in murine model. (**a**) M-mode of parasternal long axis view. White arrow: PST in the interventricular septum. (**b**) Tissue Doppler Imaging (TDI) of the parasternal long axis view. Green hollow arrows: PST in the interventricular septum (IVS). (**c**) example of PST in patient with MFS. M-mode of the parasternal long axis view. Green hollow arrows: PST in the IVS.

The presence of PST was assessed in the offline M-mode analysis of the basal septum from the parasternal long axis view. It was defined by the presence of a “double peak sign”, consisting of a normal shaped deformation pattern (first peak) during the ejection period, followed by an ongoing deformation (second peak) after aortic valve closure and within the isovolumic relaxation time period in the entire recording with at least 10 beats (AVC) (Fig. 1a). AVC was indicated manually from the M-mode of the aortic valve movement in the parasternal long axis view. In the murine model, the reproducibility of PST presence at the basal septum was confirmed and assessed using color-coded tissue Doppler images (TDI) from the parasternal long axis view in randomly chosen MFS (n = 21) and WT (n = 14) mice (Fig. 1b). The agreement between M-mode and TDI measurements was assessed by the Kappa statistics.

In humans, the presence of PST was similarly assessed in M-mode recordings obtained with the cursor positioned in the LV basal septum in a parasternal long axis view. A subject was considered to have PST if a “double peak sign” was consistently identified in the entire recording with at least 3 beats. In the human group, PST was assessed in a similar way, considered present if the PST curve was 5% bigger than systolic thickening curve staring the metric measurement [mm] from the baseline of interventricular septum in the end-diastole phase^14^ (Fig. 1c). In humans PST was not assessed using color-coded tissue Doppler images (TDI) because of low signal-to-noise ratio.

### Statistical analysis

Continuous variables were expressed as mean ± standard deviation (SD). Assessment of normal distribution was performed using the Kolmogorov–Smirnov test or Shapiro–Wilk test, depending on the size of the group assessed. Categorical variables were expressed as percentages and absolute values. To assess the presence of any difference between MFS and WT groups, the two-sample *t*-test (adjusting for unequal variances) was used to compare continuous variables if normally distributed and the Mann–Whitney U test was used otherwise. Categorical variables were compared using the χ^2^ test. The inter-observer and intra-observer reproducibility of PST values was assessed using Cohen’s Kappa statistics on ten randomly selected cases, considering Kappa values < 0.2 as poor, 0.2–0.4 as fair, 0.4–0.6 as moderate, 0.6–0.8 as good, and 0.8–1.0 as very good concordance. Statistical significance was set at 5% for all the tests. The statistical analysis was conducted using SPSS version 21.0 (SPSS Inc., Chicago, IL, USA).

## Results

### PST presence in a mouse model of MFS

Table 1 shows the characteristics of the 4 mo-age mice, while those from the 9 mo-age mice are shown in the Supplementary Table 1 (Table 1S). No differences were found in weight, gender or heart rate between MFS and WT animal groups (Table 1 and 1S).

**Table 1:** Left ventricular and aortic remodeling in 4 mo-age mice.

	WT N = 21	MFS N = 18	P
Male gender[%]	48	50	0.88
Weight [g]	24.3 ± 2.8	25.7 ± 3.5	0.16
AoR [mm]	1.48 ± 0.11	1.84 ± 0.12	** < 0.001**
LVEDD [mm]	3.75 ± 0.38	3.96 ± 0.31	0.11
LVESD [mm]	2.54 ± 0.38	2.73 ± 0.27	0.06
LV EF [%]	61 ± 7	59 ± 6	0.36
IVS [mm]	0.59 ± 0.07	0.64 ± 0.05	**0.01**
PW [mm]	0.57 ± 0.07	0.63 ± 0.07	**0.02**
LV mass [mg]	56.9 ± 17	68.7 ± 16	**0.02**
SBP [mmHg]	130 ± 11	134 ± 11	0.6
DBP [mmHg]	79 ± 9	78.7 ± 7	0.99
HR [bpm]	364 ± 41	360 ± 43	0.74

*AoR* aortic root diameter, *LVEDD* left ventricular end-diastolic diameter, *LVESD* left ventricular end-systolic diameter, *LVEF* left ventricular ejection fraction, *IVS* interventricular septum thickness, *PW* posterior wall thickness, *SBP* systolic blood pressure, *DBP* diastolic blood pressure, *HR* heart rate.

Continuous variables data is presented as mean ± SD. Independent two-sample t-test for normally distributed continuous variables and the Mann–Whitney U test for non-parametric distribution. Categorical variable (male gender) is presented as percentage (%) and compared using χ^2^ test. P<0.05, in bold, denote statistical significance.

Among the 9 mo-age mice, the aortic and LV dimensions were significantly larger in MFS mice than they were in WT mice, while LV performance, assessed through the LV ejection fraction, was lower in the MFS mice. In the 4 mo-age mice, the aortic dimensions were larger, and LV walls thicker, in MFS mice as compared to those observed in the WT mice. This was in agreement with the observations from the 9 mo-age mice, although no differences in LV chamber dimensions or function were present in the 4 mo-age mice (both MFS and WT mice showed similar LV ejection fraction). No significant differences in blood pressure were observed between MFS and WT animals at the 4 mo-age (Table 1).

**Figure 2:**
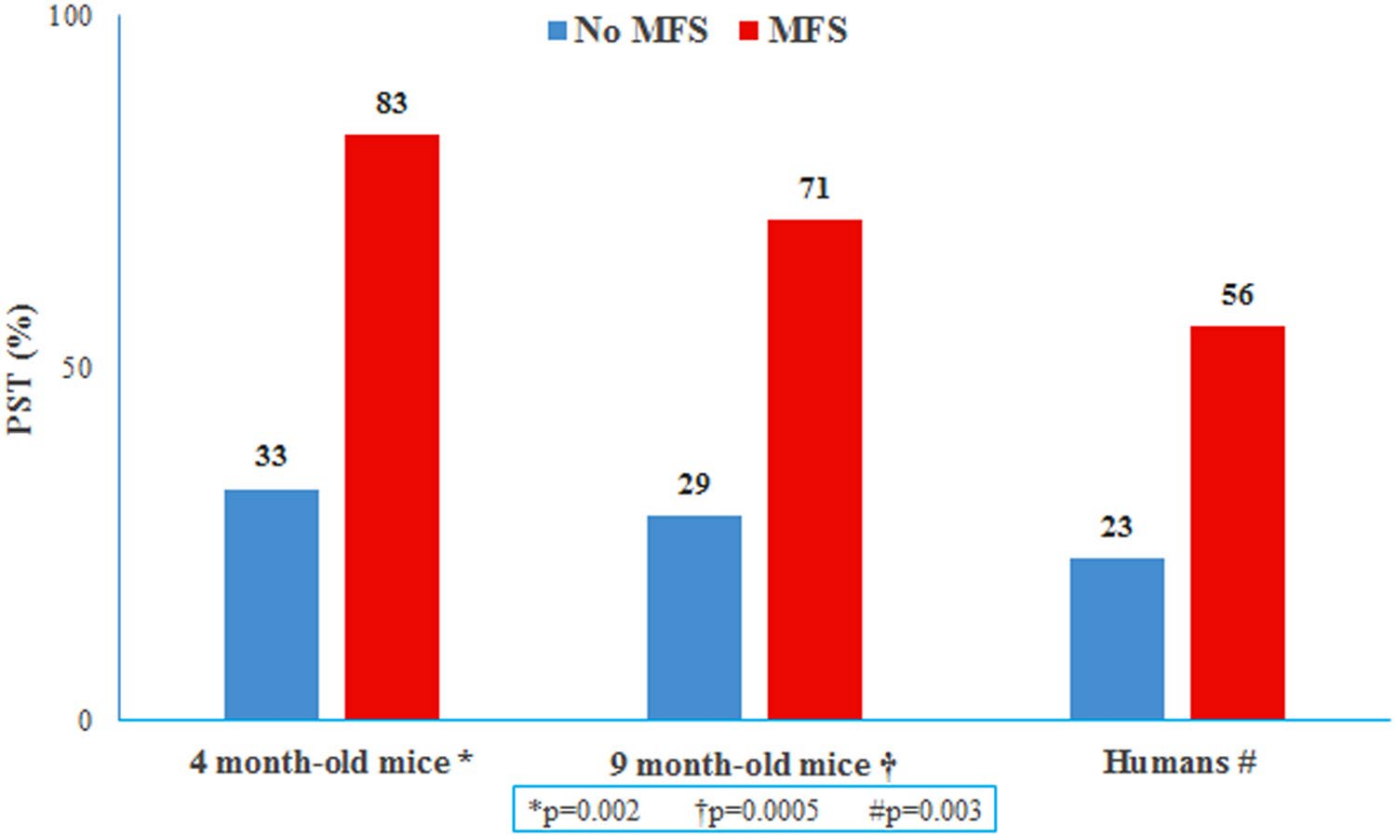
PST + prevalence in 4 month-old mice, 9 month-old mice and humans comparing MFS and No MFS groups. Data presented as percentage (%). Compared using the χ^2^ test. Significance P < 0.005.

PST was present in both MFS and WT mice, but it was significantly more frequent in MFS than it was in WT mice (Fig. 2). A high concordance was found between M-mode and TDI methods to detect the presence of PST (kappa value = 0.77) in the murine population (kappa 0.85; p < 0.01)**.** Inter-observer reliability was high for the measurement of PST (M-mode assessment: kappa = 0.78, P = 0.011; TDI assessment: kappa = 0.8 P = 0.01) in the murine model.

### PST presence in human patients with MFS

Table 2 depicts the characteristics of the clinical group regarding left ventricular and aortic remodeling. Subjects with MFS showed higher body surface area and height than did controls (2.05 vs 1.88 P < 0,001 and 187 vs 175 P < 0,001 respectively). As expected, the aorta was larger in patients with MFS than in controls. Although falling within the normal range, the left ventricular ejection fraction was lower in patients with MFS as compared to healthy controls. Conversely, systolic blood pressure was higher in healthy control subjects than in subjects with MFS (126 vs 119 mmHg, P = 0,009). Thirty patients (67%) with MFS were receiving blood pressure lowering treatment. Half of the patients (N = 23, 51%) received beta blockers and 29% losartan (N = 13), 6 were on both drugs and 33% (N = 15) received no treatment. Despite having even lower blood pressure, the presence of PST was also significantly more frequent in MFS patients as compared to healthy controls: prevalence of PST was 56% vs 23%, respectively (p = 0.003) (Fig. 2).

**Table 2:** Left ventricular and aortic remodeling in humans.

	Controls N = 35	MFS N = 45	P
Age [yo]	32 ± 7.8	29 ± 7,6	0.09
Male gender [%]	41	46	0.63
Height [cm]	175 ± 11	187 ± 11	** < 0,001**
Weight [kg]	72 ± 12	80 ± 15	**0.02**
BSA [m^2^]	1.88 ± 0.19	2.05 ± 0.2	< **0.001**
AoR ind. [mm/m^2^]	16.4 ± 2	20.7 ± 2.9	** < 0.001**
Asc Ao ind. [mm/m^2^]	14.4 ± 1.64	16.2 ± 2.34	**0.001**
LVEDD ind. [mm/m^2^]	26.6 ± 2.4	25.7 ± 3.6	0.22
LVESD ind. [mm/m^2^]	17.4 ± 1.5	16.2 ± 3.1	0.05
IVS ind. [mm/m^2^]	4.6 ± 0.7	4.9 ± 0.8	0.12
PW ind. [mm/m^2^]	4.1 ± 0.6	3.9 ± 0.4	0.13
LV EF [%]	64 ± 5	61 ± 5	**0.01**
SBP [mm/Hg]	126 ± 10	119 ± 12	**0.00**
DBP [mm/Hg]	76 ± 10	72 ± 9	0.15

*BSA* body surface area, *AoR* aortic root diameter, *Asc Ao* ascending aorta diameter, *LVEDD* left ventricular end-diastolic diameter, *LVESD* left ventricular end-systolic diameter, *LVEF* left ventricular ejection fraction, *IVS* interventricular septum thickness, *PW* left ventricular posterior wall thickness, *SBP* systolic blood pressure, *DBP* diastolic blood pressure, *ind.* indexed by BSA.

Continuous variables data is presented as mean ± SD. Independent two-sample t-test for normally distributed continuous variables and the Mann–Whitney U test for non-parametric distribution. Categorical variable (male gender) is presented as percentage (%) and compared using χ^2^ test. P<0.05, in bold, denote statistical significance.

### Association of PST with aorta dilation

Table 3 shows the differences in 4 mo-age mice with and without PST. Aortic root diameter was larger in MFS mice with PST than it was in those without, despite showing similar blood pressures. Similar results were also observed in 9 mo-age mice, with larger aortas in those with PST despite blood pressure not being assessed (Table 2S).

**Table 3:** Left ventricular and aortic remodeling in the 4 mo-age mice according to the presence of PST.

	WT N = 21		P	MFS N = 18		P
	PST (−) N = 14	PST ( +) N = 7		PST (-) N = 3	PST ( +) N = 15	
Weight [g]	**23.05** ± **2.36**	**26.94** ± **1.5**	**0.001**	22.9 ± 3	26.2 ± 3.4	0.13
AoR [mm]	1.485 ± 0.12	1.48 ± 0.09	0.92	**1.69 ± 0.06**	**1.88 ± 0.1**	**0.01**
LVEDD [mm]	3.65 ± 0.35	3.96 ± 0.38	0.17	4 ± 0.3	3.95 ± 0.3	0.82
LVESD [mm]	2.45 ± 0.34	2.75 ± 0.42	0.12	2.85 ± 0.1	2.7 ± 0.3	0.43
LVEF [%]	62 ± 8	60 ± 7	0.48	56 ± 6	60 ± 6	0.57
IVS [mm]	0.58 ± 0.08	0.6 ± 0.06	0.54	0.64 ± 0.06	0.64 ± 0.05	0.82
PW [mm]	0.56 ± 0.07	0.61 ± 0.07	0.13	0.64 ± 0.05	0.62 ± 0.07	0.74
LV mass [mg]	53 ± 15	65 ± 19	0.2	71 ± 16	68 ± 17	0.8
HR [bpm]	362 ± 45	368 ± 37	0.75	342 ± 17	363 ± 45	0.19
SBP [mm/Hg]	131 ± 11	129 ± 11	0.66	133 ± 11	134 ± 11	1
DBP [mm/Hg]	78 ± 10	81 ± 8	0.43	77,5 ± 9	79 ± 6	0.9

*AoR* aortic root diameter, *LVEDD* left ventricular end-diastolic diameter, *LVESD* left ventricular end-systolic diameter, *LVEF* left ventricular ejection fraction, *IVS* interventricular septum thickness, *PW* posterior wall thickness, *HR* heart rate, *SBP* systolic blood pressure, *DBP* diastolic blood pressure.

Data is presented as mean ± SD. Independent two-sample t-test for normally distributed variables and the Mann–Whitney U test for non-parametric distribution. SP<0.05, in bold, denote statistical significance.

Table 4 depicts human subjects’ characteristics according to the presence of PST. In the study’s human group, the presence of PST was only related to the subjects’ older age. There was no difference determined by the PST presence in the aorta dimensions or blood pressure.

**Table 4:** Left ventricular and aortic remodeling in humans according to the presence of PST.

	Controls N = 35		P	MFS N = 45		P
	PST (−) N = 27	PST ( +) N = 8		PST (-) N = 20	PST ( +) N = 25	
**Age [yo]**	31 ± 7	35 ± 9	0.21	**25 ± 7**	**31 ± 7**	**0.01**
Male gender [%]	44	50	0.78	55	48	0.64
BSA [m^2^]	1.9 ± 0.2	1.9 ± 0.2	0.9	2,07 ± 0,2	2.03 ± 0.2	0.46
AoR ind. [mm/m^2^]	16.6 ± 2	15.9 ± 2.2	0.44	20.6 ± 3	20.8 ± 2.9	0.93
Asc Ao ind. [mm/m^2^]	14.3 ± 1.6	14.8 ± 1.9	0.5	16.6 ± 2.8	15.9 ± 1.9	0.66
LVEDD ind. [mm/m^2^]	26.6 ± 2.3	26.6 ± 2.9	0.98	25.9 ± 3.6	25.5 ± 3.8	0.68
LVESD ind. [mm/m^2^]	17.2 ± 1.5	17.7 ± 1.6	0.41	16.4 ± 2.8	16.1 ± 3.3	0.56
IVS ind. [mm/m^2^]	4.6 ± 0.7	4.6 ± 0.5	0.8	4.9 ± 0.9	4.8 ± 0.8	0.7
PW ind. [mm/m^2^]	4.1 ± 0.6	4 ± 0,6	0.88	3.9 ± 0.5	3.9 ± 0.4	0.82
LV mass ind. [g/m^2^]	73 ± 17	73 ± 19	0.97	85.9 ± 18	79.8 ± 18	0.22
LVEF[%]	65 ± 4	62 ± 5	0.09	61 ± 6	61 ± 5	0.73
SBP[mm/Hg]	125 ± 10	127 ± 10	0.55	119 ± 10	119 ± 13	0.95
DBP[mm/Hg]	74 ± 11	80 ± 9	0.15	72 ± 8	73 ± 10	0.57

*BSA* body surface area, *AoR* aortic root diameter, *Asc Ao* ascending aorta diameter, *LVEDD* left ventricular end-diastolic diameter, *LVESD* left ventricular end-systolic diameter, *LVEF* ejection fraction, *IVS* interventricular septum thickness, *PW* posterior wall thickness, *SBP* systolic blood pressure, *DBP* diastolic blood pressure, *ind.* indexed by BSA.

Continuous variables data is presented as mean ± SD. Independent two-sample t-test for normally distributed continuous variables and the Mann–Whitney U test for non-parametric distribution. Categorical variable (male gender) is presented as percentage (%) and compared using χ^2^ test. P<0.05, in bold, denote statistical significance.

As PST has been described in older healthy individuals^14^, we focused on the youngest group of MFS subjects, from the first age tertile of the MFS population (under 24 years old, N = 16). In this MFS patient subgroup, PST was indeed associated with larger aortic dimensions: 19.4 ± 3 vs 15.5 ± 3 in PST ( +) vs PST (−) youngest MFS, respectively ([mm/m^2^], P = 0.04). Blood pressure was similar in the two groups of youngest MFS patients, both without PST (n = 11) and with PST (n = 5): 115 ± 9 vs 106 ± 10 ([mmHg], p = 0.07) for SBP and 69 ± 7 vs 63 ± 9 ([mmHg], p = 0.17) for DBP.

### Impact of blood pressure lowering therapy

None of the healthy controls received blood pressure lowering treatment. Conversely, 67% of patients with MFS were on medical therapy with losartan or beta-blockers. Age, blood pressure and LV cavity dimensions were similar in those with and without medication, but the aortic root was larger in those receiving medical treatment. Finally, those two groups of patients with MFS had a similar prevalence of PST (Table 5). Because of the transversal nature of the study we have no data on the duration of the medical treatment in each patient.

**Table 5:** Left ventricular and aortic remodeling in humans according to medical treatment.

	MFS without drugs N = 15	MFS with drugs N = 30	P
Age [yo]	27 ± 8	30 ± 8	0.33
Weight [kg]	76 ± 14	81 ± 15	0.26
High [cm]	183 ± 9	189 ± 12	0.08
BSA [m^2^]	1.97 ± 0.2	2.08 ± 0.2	0.12
Aortic root ind. [mm/m^2^]	19.5 ± 3	21.3 ± 3	**0.04**
Asc Ao ind. [mm/m^2^]	16 ± 3	16 ± 2	0.95
LVEDD ind. [mm/m^2^]	27 ± 5	25 ± 3	0.13
LVESD ind. [mm/m^2^]	17 ± 4	16 ± 2	0.18
IVS ind. [mm/m^2^]	4.6 ± 1	5 ± 0,7	0.09
PW ind. [mm/m^2^]	3.9 ± 0.4	3.9 ± 0.4	0.56
LV mass ind. [g/m^2^]	81 ± 20	83 ± 18	0.63
LVEF[%]	62 ± 5	61 ± 6	0.66
SBP [mmHg]	120 ± 12	118 ± 11	0.49
DBP [mmHg]	74 ± 9	72 ± 9	0.52
PST +	8 (53%)	17 (57%)	0.832

*BSA* body surface area, *AoR* aortic root diameter, *Asc Ao* ascending aorta diameter, *LVEDD* left ventricular end-diastolic diameter, *LVESD* left ventricular end-systolic diameter, *LVEF* left ventricular ejection fraction, *IVS* interventricular septum thickness, *PW* posterior wall thickness, *SBP* systolic blood pressure, *DBP* diastolic blood pressure, PST: postsystolic thickening, ind.: indexed by BSA. Data is presented as mean ± SD for continuous variables.

Independent two-sample t-test for normally distributed continuous variables and the Mann–Whitney U test for non-parametric distribution were used. Categorical variable (PST) is presented as percentage (%) and compared using χ^2^ test. P<0.05, in bold, denote statistical significance.

## Discussion

The study’s main finding was the increased prevalence of PST in MFS. These findings were detected both in a murine experimental model and in a cohort of patients with MFS. In humans we have set the presence of PST at the level of 5% bigger than the systolic curve in order to detect myocardial dysfunction in early stage of the disease despite the majority of experiments differentiate the physiologic PST from pathologic PST if it is approximately 10–20% bigger than the systolic curve^8,15^. In the controlled murine group of 4 mo-age the PST presence was associated with the aortic root dilation despite no difference in blood pressure. Interestingly, the MFS human subjects show lower systolic blood pressure than the controls and association of PST with the age. Due to the natural increasing stiffness of the aorta with aging, the presence of PST has been described to increase in the elder population^15,25^. Accordingly, and in order to avoid, at least to some extent, the potential influence of aging on PST prevalence, the youngest tertile (18–24 years-old) of our group of MFS patients was selected. Notably, humans under 24 years of age would seem to be the most comparable, in terms of age, with the 4 mo-age mice group, considering the usual life-span of mice (18–20 mo-age.). Therefore, we sought to analyze the presence of PST and its relationship with blood pressure and aortic dilation in that young adult individuals. Notably, despite the small number of subjects (N = 16, with 5 subjects having PST +), a significant association between the presence of PST (PST +) and aortic dilation was observed in the youngest patients with MFS. Both groups of young subjects (young MFS mice and young patients) showed the same association between PST and aortic root dilation. With aging, other confounding factors might influence aortic wall stress potentially explaining that this association was not observed in older subjects with MFS. Further longitudinal and controlled clinical studies will shed more light on this potential association.

Several investigators have demonstrated that dilation of the aortic root and ascending aorta is accompanied by LV dilation and impaired left ventricular function in MFS mice^16^ as well as in humans^17^. The myocardial involvement in MFS includes impaired biventricular deformation with a subsequent reduction in long axis systolic function, as well as biventricular and atrial mild diastolic dysfunction. Altogether this has raised the hypothesis of the existence of a primary cardiomyopathy in MFS^18^. Patients with MFS can potentially develop myocardial injury such as fibrosis and myocardial architecture disorder caused by abnormal collagen deposition and organization. Tae et al. reported that the early remodeling expressed as myocardial hypertrophy in 2- and 4 mo-age MFS mice was not associated with hemodynamic overload (as valvular insufficiency), but likely triggered by an intrinsic mechanism related to the altered myocardial matrix causing persistent mechanical stress^19^. In an experiment with mild transverse aortic constriction, Rouf et al. demonstrated that *Fbn1*^*C1039G/*+^ mice are more predisposed to load-induced heart failure than their WT littermates^20^. Likewise, they support the hypothesis previously reported that the canonical TGF-β signaling contributes to this load-induced cardiac decompensation^16^. An interesting novel in vivo mouse model of Cavanaugh et al. consisting of subcutaneously delivered angiotensin II in MFS mice causes accelerated aortic aneurysm formation and dilated cardiomyopathy even without aortic insufficiency, suggesting a potential intrinsic aetiology for the diseased myocardium^21^.

PST is an easy parameter to be clinically and experimentally obtained and evaluated. The data included in this study can demonstrably be reproduced using two different methodologies: DTI and M-mode echocardiography. PST in the basal septum is a marker of pressure overload that can be easily detected by conventional echocardiography as several clinical study show in hypertensive and ischemic patients^7,11^. According to the geometry of the LV, the greatest pressure overload can be expected to occur in the basal segment of the IVS as it has a greater local radius of anatomic curvature in comparison with the free wall or the apex of the LV. A regional increase in parietal stress occurs at that location, resulting both in segment interaction and PST. In the event of pressure overload, the basal IVS segment is the first parameter to evidence the associated systolic deformation reduction due to its flatter geometry^15,22^. The development of postsystolic deformation, along with localized hypertrophy, can even be observed before visual assessment of wall thickening. Previous studies have shown an association of PST with more advanced left ventricular and atrial dysfunction among patients with well-controlled systemic hypertension^23^.

From the observations gathered on LV remodeling, PST, and its association with aortic dilation in very young subjects, we would conclude that individuals with MFS show the same sign that is observed in hypertensive cardiac remodeling despite having normal blood pressures. There are two possible explanations. First, the potentially abnormal arterial wall composition in MFS, and its associated stiffening, would alter flow propagation and pressure wave reflections, thus impacting the LV performance, even though peripheral blood pressure values remain within normal ranges. The mechanism of this effect remains still not elucidated. Second, extracellular matrix assembly alterations caused by mutated fibrillin-1, altered TGFβ signaling, and associated cardiac fibrosis^24^ altogether contribute to the local wall stress mainly on cardiomyocytes, finally leading to pressure overload signs such as high LV mass and PST. Our findings suggest that the LV myocardial tissue of patients with MFS is at risk despite these patients having ‘normal blood pressures’, and would encourage searching for novel preventive therapies especially at very young age.

## Clinical implications

Preventing aortic dilation and rupture in patients with hypertension and aortic dilation is of vital importance and the benefits of vasodilator drugs in this scenario are well established. However, the role of vasodilation in preventing aortic remodeling in MFS without classical hypertension remains unclear^26^. While agents such as losartan have a vasodilator effect, its cardiovascular impact likely also occurs via non-hemodynamic modulation of TGFβ and fibrotic pathways, which has been evidenced in multiple animal studies but in clinics is much less known. Unfortunately, our study does not provide new data in this respect. Nevertheless, the ultrasound analysis of LV regional deformation patterns (as shown by PST) can easily be integrated into the clinical setting (by using M-mode or DTI) and detect signs of myocardial injury even in early stages of the disease (as in very young adults). Elucidating the mechanisms of cardiac dysfunction in MFS has important implications for the timing of surgical valve repair and for the development of novel medical therapies to prevent heart failure. The demonstration of mechanistic evidence of myocardial susceptibility in MFS heart would support the rationale for more intensive preventive therapy in still-early, asymptomatic stages of the disease, even when patients show normal peripheral blood pressure.

## Limitations of the study

The study has several strengths and limitations. Murine heart evaluation is challenging because of the smallness and high heart rate of the mice. Despite these limitations, evaluation of cardiac function has demonstrated to be feasible and reproducible in most mice following a strict methodology with high frame rate acquisitions. Despite a higher mean arterial pressure has been reported in mice using the tail cuff method as compared to the more robust methods of either intra-arterial catheter or telemetry, we chose the tail cuff measurements because of their non-invasiveness to avoid complications. The absolute number of systolic or especially diastolic blood pressure cannot be taken into account as referential for the small animals. However, we believe that it can be used to compare the values in a group of animals that are being exposed to the same conditions of assessment.

The inherent higher noise level in tissue Doppler assessment encouraged us establishing the M-mode modality as a referential of PST presence. Although we are conscious that the very sensitive level of PST detection (5% of difference) could provide some percentage of physiological examples we believe that it would not change the overall message of the study in such high difference between PST presence in MFS subjects and healthy subjects. We also acknowledge the limited sample size of the very young human population limiting the confidence of some conclusions. The transversal nature of this observational study and the absence of follow-up data make it impossible to demonstrate the impact of blood pressure lowering drugs on LV performance or whether or not these drugs can modulate the association of PST with an aortic aneurysm. The role of vasodilator therapy as a modifier of myocardial remodeling in murine MFS models is an objective of our upcoming studies. Regarding the mechanism leading to the presence of PST, data from cardiac MRI to depict myocardial fibrosis and gain a better estimation of aortic stiffness with 4D flow sequences, as well as histological studies on aorta and myocardial wall structure, will shed more light on this important topic. Further studies are needed to confirm our findings and to support our mechanistic hypothesis of an increased prevalence of PST in MFS patients. Follow-up studies are needed to evaluate the impact of vasodilator drugs on LV performance and whether or not these drugs can modulate the association of PST with an aortic aneurysm.

## Conclusions

The prevalence of PST is significantly increased both in MFS mice and patients, suggesting early LV damage. It provides a potential diagnostic tool to monitor and potentially to interfere with cardiac programming. This observation suggests the suitability of initiating early preventive therapy in patients with MFS even having normal blood pressure levels. In any case, further prospective and controlled studies are needed to examine the impact of vasodilator therapy and particularly its titration, on PST and myocardial function and aortic stiffness in patients with MFS. Mice models represent also a perfect controlled experimental setting to prove this, once that we have shown in the current study that PST measurement is feasible and reproducible in them.

## Supplementary Information

Supplementary Information.
