# Supplementary material for: Postsystolic thickening is a potential new clinical sign of injured myocardium in marfan syndrome
Source: Sci Rep. 2021 Aug 4;11:15790. doi: 10.1038/s41598-021-95263-5 (PMC8338999; doi:10.1038/s41598-021-95263-5)
Supplement: Supplementary file 1 — Supplementary Information. [file 41598_2021_95263_MOESM1_ESM.docx]

**POSTSYSTOLIC THICKENING IS A POTENTIAL NEW CLINICAL SIGN OF INJURED MYOCARDIUM IN MARFAN SYNDROME**

*Aleksandra Mas-Stachurska 1, 2 (MD, MSc), Gustavo Egea 3 (PhD), Rianne de Bruin-Bon 4, Paula Rudenick 5 (PhD), Laura Sanchis 1 (MD, PhD), Berto J. Bouma 4 (MD, PhD), Barbara J. Mulder 4 (MD, PhD), Bart Bijnens 1, 5 (PhD), Marta Sitges 6 (MD, PhD)

1 Cardiovascular Institute, Hospital Clinic, University of Barcelona and Institut d’Investigacions Biomèdiques August Pi i Sunyer (IDIBAPS); CERCA Programme/Generalitat de Catalunya, Barcelona, Spain

2 Institut Hospital del Mar d'Investigacions Mèdiques (IMIM), Barcelona, Spain

3 Department of Biomedical Sciences, University of Barcelona School of Medicine and Health Sciences and Institut d’Investigacions Biomèdiques August Pi i Sunyer (IDIBAPS), Barcelona, Spain

4 Cardiology Department, Amsterdam University Medical Centers, Location Academic Medic Centrum, Amsterdam, Netherlands.

5 ICREA, Barcelona, Spain.

6 Cardiovascular Institute, Hospital Clinic, University of Barcelona School of Medicine and Health Sciences and Institut d’Investigacions Biomèdiques August Pi i Sunyer (IDIBAPS), CIBERCV, Instituto de Salud Carlos III (CB16/11/00354); CERCA Programme/Generalitat de Catalunya, Spain.

**Bart Bijnens and Marta Sitges both equally contributed and are both considered senior authors.**

*Corresponding author: Dr Aleksandra Mas-Stachurska.

Address for correspondence: Cardiology Department, Hospital del Mar, Passeig Maritim 25-29, 08003 Barcelona, Spain. [astachur@gmail.com](mailto:astachur@gmail.com)

Key words: Marfan syndrome, aneurysm, aorta, cardiomyopathy, vasodilator

**SUPPLEMENTAL MATERIAL (EXPANDED RESULTS)**

|  | **WT**  **N=55** | **MFS**  **N=53** | **P** |
| --- | --- | --- | --- |
|  |  |  |  |
| weight [g] | 27.54 ± 3.69 | 27.59 ± 2.87 | 0.967 |
| AoR [mm] | 1.63 ± 0.15 | 2.06 ± 0.16 | **<0.001** |
| LVEDD [mm] | 3.80 ± 0.33 | 3.98 ± 0.29 | **0.002** |
| LVESD [mm] | 2.54 ± 0.17 | 2.76 ± 0.30 | **0.005** |
| LV EF [%] | 67 ± 6 | 64 ± 5 | **0.01** |
| IVS [mm] | 0.69 ± 0.06 | 0.73 ± 0.07 | **0.001** |
| PW [mm] | 0.68 ± 0.07 | 0.72 ± 0.07 | **0.008** |
| HR [bpm] | 389.11 ± 43.67 | 394.22 ± 47.64 | 0.62 |

**Table 1S.** Left ventricular and aortic remodeling in 9 mo-age mice. AoR: aortic root diameter, LVEDD: left ventricular end-diastolic diameter, LVESD: left ventricular end-systolic diameter, LV EF: left ventricular ejection fraction, IVS: interventricular septum thickness, PW: posterior wall thickness, HR: heart rate. Data is presented as mean ± SD. Independent two-sample t-test for normally distributed variables and the Mann-Whitney U test for non-parametric distribution. Significance: p<0.05.

|  | **WT**  **N=55** | | **P** | **MFS**  **N=53** | | **P** |
| --- | --- | --- | --- | --- | --- | --- |
|  | **PST (-)**  **N=39** | **PST (+)**  **N=16** |  | **PST (-)**  **N=15** | **PST (+)**  **N=38** |  |
| AoR [mm] | 1.61 ± 0.12 | 1.80 ± 0.22 | **0.004** | 1.95 ± 0.15 | 2.13 ± 0.13 | **< 0.0005** |
| LVEDD [mm] | 3.65 ±0.80 | 3.88 ± 0.53 | 0.66 | 3.91 ± 0.29 | 3.97 ± 30 | 0.53 |
| LVESD [mm] | 2.48 ± 0.59 | 2.66 ± 0.48 | 0.4 | 2.72 ± 0.23 | 2.75 ± 0.29 | 0.77 |
| LV EF [%] | 66 ± 6 | 66 ± 6 | 0.88 | 63 ± 4 | 64 ± 5 | 0.48 |
| IVS [mm] | 0.67 ± 0.15 | 0.7 ± 0.09 | 0.64 | 0.73 ± 0.08 | 0.72 ± 0.06 | 0.73 |
| PW [mm] | 0.64 ± 0.15 | 0.7 ± 0.12 | 0.27 | 0.73 ± 0.07 | 0.71 ± 0.07 | 0.57 |
| HR [bpm] | 3.96 ± 46 | 372 ± 33 | 0.15 | 389 ± 56 | 396 ± 45 | 0.66 |

**Table 2S.** Left ventricular and aortic remodeling according to the presence of PST in 9 mo-age old mice. AoR: aortic root diameter, LVEDD: left ventricular end-diastolic diameter, LVESD: left ventricular end-systolic diameter, LV EF: left ventricular ejection fraction, IVS: interventricular septum thickness, PW: posterior wall thickness, HR: heart rate. Data is presented as mean ± SD. Independent two-sample t-test for normally distributed variables and the Mann-Whitney U test for non-parametric distribution. Significance: p<0.05.
